# The influence of electromagnetic radiation of cell phones on the behavior of animals

**DOI:** 10.14202/vetworld.2020.549-555

**Published:** 2020-03-24

**Authors:** Innar Sultangaliyeva, Raikhan Beisenova, Rumiya Tazitdinova, Akhan Abzhalelov, Marat Khanturin

**Affiliations:** 1 Department of Management and Engineering in the Field of Environmental Protection, L.N. Gumilyov Eurasian National University, Nur-Sultan, Kazakhstan; 2 Department of Geography, Ecology and Tourism, Sh. Ualikhanov Kokshetau State University, Kokshetau, Kazakhstan

**Keywords:** cell phone, central nervous system, electromagnetic pollution, electromagnetic radiation, locomotion

## Abstract

**Background and Aim:**

The radiation emitted from cell phones has various deleterious effects on human health. The article considers the problem of the effects of electromagnetic radiation (EMR) of cell phones on the behavior of animals. The use of mobile phones is continually increasing throughout the world. All of the world population uses mobile phones widely; thus, having strong and daily effect on the brain. There is no restriction on the use of mobile phones. Getting more advantages and convenience of mobile communication, the population is trying not to hear the information about possible risks to their health, there is no element of self-restraint. The aim of this work was to study behavioral patterns in response to the influence of cell phones.

**Materials and Methods:**

The experiments were carried out on 90 white outbred rats weighing 250-300 g. Experiments were carried out and the effect of EMR from Samsung Galaxy J1 mini and Xiaomi Redmi S2 phones on animal behavior was studied. Behavioral reactions were studied using the open field method.

**Results:**

By the quality of the act of locomotion in the second experimental group is reduced by 30% and also in this group by the time of the act is 23% lower than in the control data. Vertical motor activity, a support stand, is also suppressed in the second experimental group: 61% lower in number and 47.2% lower in act time compared to the control group. Based on the data, we can conclude that the EMR of the phone Samsung Galaxy J1 Mini affects the behavior of animals to a greater extent than Xiaomi Redmi S2. By the frequency of the act, sniffing is 26% lower; by the time of the act, it remained at the level of control data in the second group. In the third group of the animals, the number and time of the act are 15% lower than the control data. The support stands as an indicator of research activity. In the second group the act of stand with support – by number was lower by 57% than the control data, this indicates that research activity is being suppressed.

**Conclusion:**

According to the above changes under the action of EMR from the Samsung Galaxy J1 Mini and Xiaomi Redmi S2 phones, the motor component of behavior is suppressed. The research component of behavior in all groups of animals is enhanced due to high rates of sniffing. It is possible that in animals under stress, the motivation to interact with the environment decreases, leading to an excessive self-accentuation of the animal.

## Introduction

An increasingly growing role in the overall flow of negative anthropogenic impacts on the biosphere is acquired by physical pollution. This is due to changes in physical parameters of the environment, that is, with their deviation from the natural background. Recently, there has been rapid electromagnetic pollution (EMP) of the environment [1]. The World Health Organization (WHO) pays special attention to the problem of negative effects from exposure to electromagnetic fields (EMF). WHO has included the problem of EMP of the environment among the priority issues of humanity [2]. It is expected that technological growth will continue, in connection with this, the influence of EMF will continue to grow steadily. All sources of EMF, as a rule, are a provenance of complex electromagnetic radiation (EMR), which affects plants, animals, insects, and soil flora in the zone of influence of EMF [3]. Their levels in some cases exceed the maximum recorded natural electromagnetic background by 200-30,000 times. Strong deviations of the EMF from the natural level, to a greater or lesser extent, go beyond the limits of the vital activity of living organisms and are a stress factor [4]. Among the sources of anthropogenic effects of EMR on the population should be allocated cell phones. EMR generated by mobile communications acquires the role of a permanent source of environmental pollution [5-7]. The question of the nature of the influence of the EMF on the human body and its functions remains debatable. Disputes about the dangers or harmlessness of mobile phones are ongoing, and at present, they are based on a huge number of experimental and theoretical studies published in leading international scientific journals. In a study of Kim *et al*. [8], it was found that EMFs can cause changes in the nerve cells of the central nervous system, including apoptosis of neuronal cells, changes in the nerve myelin, and ion channels; moreover, radiofrequency (RF) EMFs act as a source of stress in living beings. In a review of the work, the authors summarize the possible biological effects of exposure to RF EMFs. As a possible mechanism for changing neurological functions when exposed to EMFs, the authors are confident that more mechanisms will be involved than mentioned, but they also summarized only recent studies of thermal effects, activation of autophagy, and changes in the ion channel. Most of these studies were performed using cell or animal models, and basic information was provided on the possible biological effects of exposure to RF beams to RF EMFs. Thus, these results can not relate to people directly [8]. To confirm the possible biological effects of exposure to EMFs on humans, physiological methods of research are needed. In the work of Vereshchako *et al*. [9], when studying the state of the reproductive system of male rats of the first generation using a cellular phone (897 MHz, daily, 8 h/day), it has been found that irradiation causes a decrease in the number of births of animals, changing the sex ratio towards the increase in the number of males. The data obtained also indicate the occurrence of significant violations in the reproductive system of male rats with prolonged exposure to EMR [9]. The study of the non-profit Ramazzini Institute (RI) carried out an experimental study to evaluate the carcinogenic effects of exposure to radiofrequency radiation (RFR) generated by base stations of mobile phones on rats. The RI performed a life-span carcinogenic study on Sprague-Dawley rats to evaluate the carcinogenic effects of RFR in the situation of far field, reproducing the environmental exposure to RFR generated by 1.8 GHz GSM antenna of the radio base stations of mobile phone. This is the largest long-term study ever performed on rats on the health effects of RFR, including 2448 animals. At the highest dose (50 V/m), the researchers found a statistically significant increase in the incidence of heart schwannomas in male rats. Moreover, they observed elevated rates of heart Schwann cell hyperplasia in male and female rats, as well as of malignant gliomas in female rats, although neither of these findings were statistically significant. The researchers concluded that the experiment provided “sufficient evidence” to call for the re-evaluation of the IARCs conclusions as to the carcinogenic potential of RFR in humans [10]. In the work of Ouadah *et al*. [11], data were proposed that suggest the possible effect of RF EMFs on brain tumors of laboratory Wistar rats by reducing the invasion of immune cells and apoptosis of glioblastoma cells, possibly due to low amplitude to affect survival [11]. Eger *et al*. [12] took up a challenge to medical professionals by Germany’s radiation protection board to determine if there was an increased cancer incidence in populations living near cell towers. Their study evaluated data for approximately 1000 patients between the years of 1994 and 2004 who lived close to cell antennas. The results showed that the incidence of cancer was significantly higher among those patients who had lived for 5-10 years at a distance of up to 400 miles from a cell installation that had been in operation since 1993. Compared with those patients living further away, and that the patients fell ill on an average of 8 years earlier than expected. In the years between 1999 and 2004, after 5 years’ operation of the transmitting installation, the relative risk of getting cancer had tripled for residents in proximity of the installation compared with inhabitants outside of the area [12]. Хu *et al*. [13] in their work investigated the effect of the 1800 MHz EMF on cell death and cell proliferation in the developing brain. The postnatal 7-day (P7) and P21 healthy Kunming mice were taken and randomly distributed into the experimental and control groups. Electromagnetic effects did not significantly affect the cell death in the dentate gyrus of the hippocampus in P7 and P21 mice, as indicated by active immunostaining of caspase-3 and labeling with fluoride nephritis. The proliferation of basal cells in the hippocampus was higher in P7 than in P21 mice, as evidenced by the number of BrdU-labeled cells and immunohistochemical staining for phosphorous histone H3 (PHH3) and brain lipid-binding protein (BLBP). Electromagnetic stimulation stimulated DNA synthesis in neural stem cells and P7 progenitor cells, but reduced cell division and the total number of stem cells in the hippocampus, as evidenced by an increase in BrdU labeling and a decrease in PHH3 and BLBP labeling in comparison with P7 control mice. There were no significant changes in cell proliferation in P21 mice after exposure to an EMF. These results show that the effect on the proliferation of stem cells with short-term exposure to an EMF of 1800 MHz depends on the stage of brain development [13]. The study of Gorshunova *et al*. [14] reported the effect of EMR of computed tomography on the aging process and the vegetative nervous system. They found that individuals affected by mobile communications are aging physiologically. However, there was a slight increase in the difference between the calendar and biological age, as well as the Bayevsky stress index (dominance of the parasympathetic nervous system) due to the duration of the use of mobile phones [14]. Abdel-Rassoul *et al*. [15] conducted a study on the neurobehavioral effects among residents around cell phone base stations. The authors found that residents living near base stations of mobile phones are at risk of developing neuropsychiatric problems and some changes in the functioning of neurobehavioral functions as a result of relief or inhibition. Therefore, it is recommended to revise the standard guidelines for exposure of the public from baseline antennas of mobile phones [15]. Based on the work of Grigoriyev and Biriukov [16] on the basis of a study of numerous experimental and epidemiological findings, it was found that EMR of mobile communication has a negative effect on public health. Analysis of the effect of RF radiation from cell phones on users’ brains has shown that this organ is most susceptible to the effects of this type of radiation, especially in children. As a result of the study, functional changes were recorded in children – cell phones, including increased fatigue (39.7%), decline in school and home performance (50.7%), weakening the sustainability of voluntary attention (productivity – 14.3%, accuracy – 19.4%), and semantic memory (accuracy – 19.4%, an increase in time – 30.1%). In addition, noticeable changes in the speed of the auditory-motor response (55.5%) and violations of phonemic perception were noted. Currently, we can say that uncontrolled use of mobile communications has potential danger to public health. The authors showed that children are at-risk and gave an assessment of the danger to children’s health. When creating a regulatory framework, it is necessary to take into account scientific ideas about the possible development of long-term brain pathologies with combined exposure to EMF, especially in children [16].

In this regard, the effect of EMR on the function of the brain is now relevant, and for experimental research, such physiological methods as the “Open field” and “Cross maze” are used, which show changes in the behavior of animals. There are two categories of animal behavior. The first includes actions performed by animals, even in complete isolation to preserve their own life and health. Such “self-directed” types of behavior can be called egocentric. The second category is social behavior, which covers actions induced or performed only in presence of individuals of their own or other types on the workof Farland [17]. The basis of various types of egocentric behavior is the need for self-preservation. These actions are associated with nutrition, removal of end products of metabolism, thirst quenching, and in species that breathe atmospheric air, even with breathing. Many of these reactions are reflex in nature and are innate, however, they are usually not attributed to instincts. With the self-preservation of the individual, actions are associated with caring for the surface of the body, especially in animals covered with wool or feathers. Such types of behavior, including grooming (fur care), preening (feather care), scratching, shaking, sipping, licking, bathing, and smearing covers, are common to all species of birds and mammals [17]. These “comfortable movements” play an important role in social behavior: on the basis of aforementioned findings, motor reactions develop, which are used as communication signals, for example, during marriage or threatening demonstrations. Foraging, i.e., food harvesting belongs to egocentric behavior. They depend on habitat characteristic of the animal, physical abilities of the individual to perform locomotor and other movements, as well as its sensory abilities, which allow detecting changes in the environment. In the course of our work, we observed directly egocentric behavior in animals [17]. Cell phone is a small-sized transceiver operating in the 900/1800 MHz range. It belongs to the open-source of EMR. Such phones currently have a large set of functions and capabilities for data transmission [18]. Xiaomi Redmi S2 is a quality smartphone with its high-resolution camera and screen. The mobile phone Samsung Galaxy J1 Mini is high-performance phone with minimal power consumption. It is the most popular series. Compared to other phones, these types of mobile phones are affordable and easily accessible among young people.

The aim of this work was to study the behavioral patterns in response to the influence of the Samsung Galaxy J1 Mini and Xiaomi Redmi S2 cell phones.

## Materials and Methods

### Ethical approval

All the experiments performed in this study were conducted in accordance with the ethical standards reflected in the following documents: The Council of international medical scientific organizations (ISMPP) has published the “Code of Ethics,” “International recommendations for conducting biomedical research using animals” (1985), and “The European Convention for the Protection of Vertebrate Animals used for Experimental and other Scientific Purposes” (Strasbourg, March 18, 1986).

### Study area

The study was conducted in the laboratory of applied Ecology of the L. N. Gumilyov Eurasian National University.

### Animals

The experiments were performed on 90 white, outbred rats. The weight of each rat was about 250-300 g. Experiments were carried out and the effect of EMR from Samsung Galaxy J1 Mini and Xiaomi Redmi S2 phones.

### Study design

This study was conducted in September 2019. Experimental animals were divided into three groups. The first group (n=30) of the control group did not receive a dose of EMR; the second group (n=30) was affected by a call to the Samsung Galaxy J1 Mini cell phone for 10 min, every 10 min; and the third group (n=30) was animals, affected by a call to the cell phone Xiaomi Redmi S2 for 10 min, every 10 min, the total exposure time was 8 h in silent mode.

The animal was placed in the center of the field, racks and locomotion (at the intersection of the applied lines) were visually recorded for 2 min.

### “Open field” (animal test) method

Behavioral reactions were studied using the open field method. “Open field” - a round area with a diameter of 150 cm, bounded by opaque sides with a height of 50 cm; the whole area of the circle was lined with lines of 16 squares, with sides of 20 cm. Experiments were carried out under natural daylight. Rats, in the field, were placed in the center of the site, then within 2 min such behavioral indicators were visually recorded as the number of crossed squares (horizontal activity), the number of stepping on the hind legs with the support of the front paws of the side and without the support (vertical activity), the number of acts of cleansing (grooming), the number of fecal boluses (defecation), and the number of acts of sniffing, movements in place, immobility, and urination.

During the experiments, the sequence and duration of each act were recorded in seconds with a stopwatch and data were recorded.

The “open field” technique, widely used in modern neurophysiological experiments, has been recognized for its simplicity and reliability. Introduced into the practice of laboratory research, it allows scientists or researchers to quantify the motor activity of mice and rats by two main components: Vertical – rack and horizontal – locomotion [19]. It is noted that quantitatively locomotion and stance and their ratio vary widely among different individuals. However, the usual increase in one component is accompanied by an increase in the other. A mathematical analysis of the relationship between these elements of the movement has not been done to date, and yet, the establishment of a statistical relationship between genetically determined forms of natural motor activity would probably indicate the presence of a certain structure that characterizes the motor behavior of the group of animals as a whole [19].

The following indicators were recorded and taken into account:

1. Motor activity: The number of squares crossed by a rat with four paws.
2. Vertical activity: The number of hind legs lifts.
3. Grooming (the number of acts of “washing”).
4. Defecation and urination: The number of fecal pellets and urine droplets deposited for the experiment.

### Statistical analysis

The presence of a relationship between the vertical and horizontal components of motor behavior was judged by the magnitude of the correlation coefficient. The results were processed by calculating the arithmetic mean (M), its average error (m), and the standard deviation (δ). To assess the reliability of differences, the student’s confidence coefficient and the probability value (P) were calculated. The statistical analysis was performed using Student’s t-test. The t-test (also sometimes called the Student’s t-test) was used to determine the significance of the difference between the means of two sets of data. In essence, the test compares the difference in means relative to the observed random variations in each set.

## Results and Discussion

The test results of the central nervous system of experimental animals, obtained during our experiments, are given in Table-1.

**Table-1:** Changes in the behavioral reactions of rats when exposed to electromagnetic radiation of the phones Samsung Galaxy J1 Mini and Xiaomi Redmi S2 phones.

Indicators	First group		Second group		Third group	
		
	Total of acts	Number of acts	Total of acts	Number of acts	Total of acts	Number of acts
Locomotion	33±0.93	14.2±0.84	25.6±0.28	8.8±0.21***	32.4±0.48	13.4±0.1
Sniffing	45.2±0.61	11.4±0.42	46.2±0.58	8.4±0.09***	38.2±0.45	9.8±0.04*
Stand with support	25±0.51	8.2±0.08	13.2±0.8*	3.2±0.05**	21.4±0.69	7.4±0.52
Standing	3.2±0.03	1.4±0.03	3±0.11	0.6±0.04**	3.4±0.01	1.6±0.22
Movement in place	8.8±0.69	4.2±0.04	11.4±0.08	3±0.06**	14.5±0.43	3.2±0.04***
Immobility	1.6	0.6±0.01	11±0.9	2.6±0.04**	4.2±0.46	0.8±0.02***
Defecation	0.4±0.02	0.2±0.02	0.4±0.03	0.2±0.01	0.6±0.04	0.2±0.01
Grooming	4.67±0.07	0.4±0.03	9.2±0.44	1.8±0.05**	7.16±0.03	2.2±0.03***

* p<0.05;

** p<0.01;

*** p<0,001 compared with the data of control animals.

The greatest number of behavioral acts falls on the act of locomotion and, accordingly, rats spend more time on it in control experiments (Table-1).

**Figure-1:**
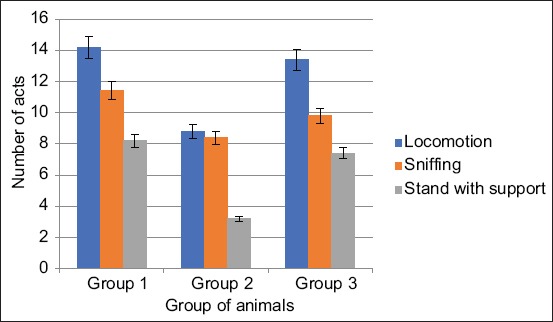
Changes in the number of acts of locomotion, sniffing, and stand with the support of animals when exposed from the Samsung Galaxy J1 Mini and Xiaomi Redmi S2 phones.

**Figure-2:**
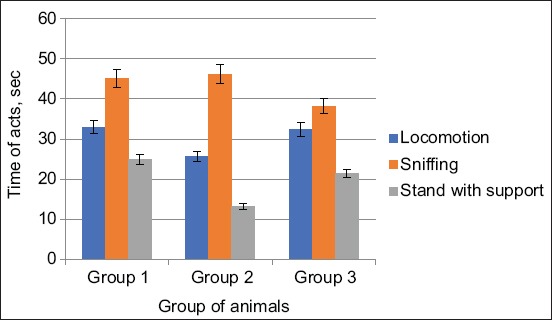
Changes in the time of acts of locomotion, sniffing, and stand with the support of animals when exposed from the Samsung Galaxy J1 Mini and Xiaomi Redmi S2 phones.

When exposed to EMR from the Samsung Galaxy J1 Mini and Xiaomi Redmi S2 phones, the nature of changes in this behavioral act was as follows: In the second group of animals, this indicator is 22.42% less in number, and the act time is 38.03% less (p<0.001). In the third group of animals, the number of this indicator is 1.8% less and the time of the act by 5.63% (Figures-1 and 2).

**Figure-3:**
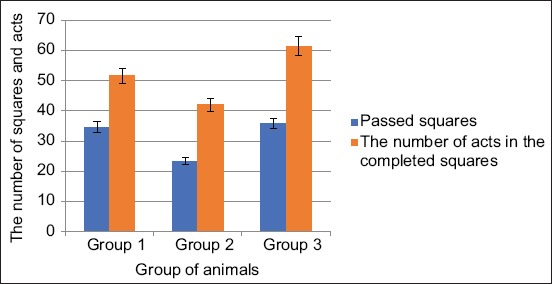
Changes in the past squares and the number of acts in the past squares when exposed from the Samsung Galaxy J1 Mini and Xiaomi Redmi S2 phones.

During locomotion, rats passed in the “open field” in the first group 34.6±0.72 squares 51.6±0.93 times, which indicates the motor activity of animals of this group. In the second group, the number of squares covered by rats was 23.4±0.77, which is 32.36% (p<0.01) below the data of the first group; in the third group, this indicator was 35.8±0.93 then there is 3.47% higher than the data of the first group (Figure-3).

These results indicate the suppression of the motor activity of animals when exposed to cell phones, but in the second group, the changes are more noticeable than in the third group of animals.

In the second group, the act of sniffing changed slightly in terms of the duration of the act and was 26.32% (p<0.001) lower in number than the same indicator of control animals. In the third group of animals, the act of sniffing by quantity is less by 14.04% (p<0.05), and the duration is 15.5% less than in the control group.

This indicates a decrease in the research activity of animals under the action of EMR from the Samsung Galaxy J1 Mini and Xiaomi Redmi S2 phones, and in the third group, the effect was more pronounced in animals.

In the second group, the first type of vertical activity was a stand with support – by number and time below control data by 47.2% (p<0.05) and 60.98% (p<0.05) than the control data. In the second type was a stand without supports – in terms of quantity and in time were lower, 57.15% lower in number (p<0.05) and slightly lower in duration than data from the control group. In the third group, the rack with quantity support was lower by 9, 76% lower and in duration 14.4% lower than the control group. Rack without support was higher by 14.28% in number and 6.25% higher than the data of the first group.

**Figure-4:**
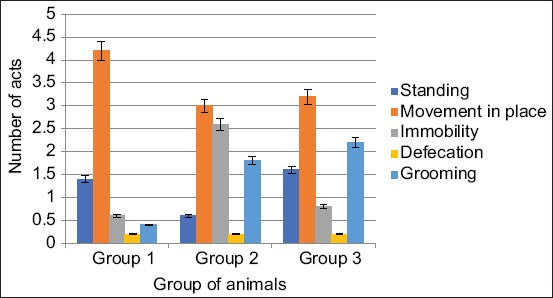
Changes in the number of acts of research and emotional activity of animals when exposed from the Samsung Galaxy J1 Mini and Xiaomi Redmi S2 phones.

**Figure-5:**
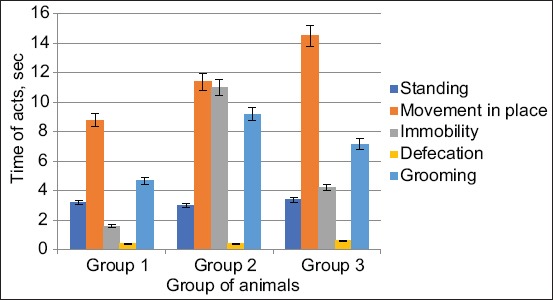
Changes in the time of acts of research and emotional activity when exposed from Samsung Galaxy J1 Mini and Xiaomi Redmi S2 phones.

In the second group, the movement in place was lower in number and in time higher than the control data by 30% (p<0.05). In the third group, the movement in place in terms of quantity was higher by 23.8% (p<0.05) lower and in duration 64.77% (p<0.001) above the control group. Rack without support was higher by 14.28% in number and 6.25% higher than the data of the first group (Figures-4 and 5).

The act of grooming in the second group of animals increased in the number of acts committed by 350% (p<0.01), and the duration of the act in this group was 97% (p<0.05) above the data of the first group. In the third group of animals, the act of grooming was 450% higher in the number (p<0.05), 53.37% in duration (p<0.05) higher than the data of the control group (Figures-4 and 5).

This indicates the strengthening of the grooming act under the action of EMR from Samsung Galaxy J1 Mini and Xiaomi Redmi S2 phones and this is probably due to the stressful activation of grooming. Stressor activation of grooming in experimental animals of the second and third groups is probably related to the adaptive need to reduce the level of arousal in animals under the action of EMR from Samsung Galaxy J1 and Xiomi Redmi S2 phones. According to other authors, the biological meaning of the “stressful” activation of grooming in rodents can be explained in terms of classical biased activity. This form of behavior is often found in rodents in the form of manipulations with one’s own body with elements of “putting oneself in order,” characterized by “a clear discrepancy between the environment in which it is implemented.”

In the second group, the act of immobility was increased in quantity by 333% (p<0.01) and in duration was higher by 587.5% (p<0.05). In the third group, the act of immobility in quantity is 33% higher and in duration higher by 162.5% (p<0.001) than in the first group. All these changes in the act of immobility indicate the suppression of the motor activity of animals when exposed to EMR from Samsung Galaxy phones J1 Mini and Xiaomi Redmi S2.

Acts of defecation in the third group in time were 50% higher than in the first group. The act of urination in experimental animals was weak in both the control and experimental groups of animals.

## Conclusion

Thus, according to the above changes, we can say that under the action of EMR from the Samsung Galaxy J1 Mini and Xiaomi Redmi S2 phones, the motor component of behavior is suppressed (horizontal – locomotion, movement in place and vertical – stand with support). Horizontal locomotor activity – locomotion is suppressed, as indicated by a decrease in the time and frequency of the act in both the experimental groups of animals, especially when exposed to Samsung Galaxy J1 Mini. By the quality of the act of locomotion in the second experimental group is reduced by 30% and also in this group by the time of the act is 23% lower than in the control data. Vertical motor activity, a support stand, is also suppressed in the second experimental group: 61% lower in number and 47.2% lower in act time compared to the control group. Based on the data, we can conclude that the EMR of the phone Samsung Galaxy J1 Mini affects the behavior of animals to a greater extent than Xiaomi Redmi S2. The research component of behavior in all groups of animals is enhanced due to high rates of sniffing. By the frequency of the act, sniffing is 26% lower; by the time of the act, it remained at the level of control data in the second group. In the third group of the animals, the number and time of the act are 15% lower than the control data. The support stands as an indicator of research activity. In the second group, the act of stand with support – by number was lower by 57% than the control data, this indicates that research activity is being suppressed. It is possible that in animals under stress, the motivation to interact with the environment decreases, leading to an excessive self-accentuation of the animal.

Immobility is seen in all experimental groups, which indicates the suppression of the emotional sphere of brain activity. Defecation and urination change only slightly, which may also indicate the suppression of animal emotions.

## Authors’ contributions

IS: Conducting experiments and data analysis. RB: Data analysis and project management. RT: Administration and editing. AA: Statistical data processing. MK: Project conclusion. All authors read and approved the final manuscript.
